# Diffusion Tensor CMR Assessment of the Microstructural Response to Dobutamine Stress in Health and Comparison With Patients With Recovered Dilated Cardiomyopathy

**DOI:** 10.1161/CIRCIMAGING.125.018226

**Published:** 2025-12-12

**Authors:** Zohya Khalique, Andrew D. Scott, Pedro F. Ferreira, Maria Molto, Sonia Nielles-Vallespin, Dudley J. Pennell

**Affiliations:** 1 Cardiovascular Magnetic Resonance Unit, Royal Brompton Hospital, Part of Guy’s and St Thomas’ NHS Foundation Trust, United Kingdom (Z.K., A.D.S., P.F.F., M.M., S.N.-V., D.J.P.).; 2 National Heart and Lung Institute, Imperial College, London, United Kingdom (Z.K., A.D.S., P.F.F., M.M., S.N.-V., D.J.P.).

**Keywords:** cardiomyopathies, dobutamine, diffusion tensor imaging, magnetic resonance imaging, myocardium, ventricular remodeling

## Abstract

**BACKGROUND:**

Contractile reserve assessment assesses myocardial performance and prognosis. The microstructural mechanisms that facilitate increased cardiac function have not been described, but can be studied using diffusion tensor cardiovascular magnetic resonance. Resting microstructural contractile function is characterized by reorientation of aggregated cardiomyocytes (sheetlets) from wall-parallel in diastole to a more wall-perpendicular configuration in systole, with the diffusion tensor cardiovascular magnetic resonance parameter E2A defining their orientation, and sheetlet mobility defining the angle through which they rotate. We used diffusion tensor cardiovascular magnetic resonance to identify the microstructural response to dobutamine stress in healthy volunteers and then compared with patients with recovered dilated cardiomyopathy (rDCM).

**METHODS:**

In this first-of-its-kind prospective observational study, 20 healthy volunteers and 32 patients with rDCM underwent diffusion tensor cardiovascular magnetic resonance at rest, during dobutamine, and on recovery.

**RESULTS:**

In healthy volunteers, both diastolic and systolic E2A increased with dobutamine stress (13±3° to 17±5°; *P*<0.001 and 59±11° to 65±7°; *P*=0.002). Sheetlet mobility remained unchanged (45±11° to 49±10°; *P*=0.19), but biphasic mean E2A increased (36±6° to 41±4°; *P*<0.001). In rDCM, diastolic E2A at rest was higher than in healthy volunteers (20±8° versus 13±3°, *P*<0.001), and sheetlet mobility was reduced (34±12° versus 45±11°; *P*<0.001). During dobutamine stress, rDCM diastolic and systolic E2A increased compared with rest (20±8° to 24±10°; *P*=0.001 and 54±13° to 63±11°; *P*=0.005). However, sheetlet mobility in patients with rDCM failed to increase with dobutamine to healthy levels (39±13° versus 49±10°; *P*=0.005).

**CONCLUSIONS:**

This is the first report describing how the myocardial microstructure facilitates cardiac reserve. In health, sheetlet mobility moves further toward the wall-perpendicular plane to drive increased contractility, rather than increased magnitude of sheetlet mobility. Despite clinical recovery in patients with rDCM, microstructural function at rest and during dobutamine remains impaired. Further understanding of microstructural remodeling at rest and during stress may help refine risk stratification of patients with rDCM at risk of relapse.

**Clinical Perspective**

Cardiac contractility at rest is mediated by sheetlet rotation from a more wall-parallel in diastole to a more wall-perpendicular systolic configuration. Beyond this, an increase in cardiac contractility is driven by swiveling the range of sheetlet rotation more radially. In patients with recovered dilated cardiomyopathy, there is persistent microstructural impairment with failure of resting diastolic sheetlet relaxation and inability to increment sheetlet mobility to healthy levels under stress. Understanding the remodeling process further on this microstructural level may help discriminate between those who maintain recovery and those at risk of relapse and support risk stratification. With the use of novel small-molecule myosin activators providing varied effects on recovery, diffusion tensor cardiovascular magnetic resonance could play a role in identifying those who may benefit from myosin activators and track drug response.

**See Editorial by Sosnovik and Nguyen**

Dilated cardiomyopathy is a common condition characterized by dilatation and impairment of the left ventricle (LV) in the absence of coronary artery disease and abnormal loading conditions.^1^ Pharmacotherapy can support reverse remodeling to improve LV size and ejection fraction (EF) to achieve remission in at least a third of patients. Predicting which patients will recover is difficult, but contractile reserve can be used as a prognostic indicator of survival and reverse remodeling.^2–8^

However, there is increasing awareness that reverse remodeling alone is not the sole determinant or definition of maintaining remission. Relapse is common in those who withdraw medical therapy, and microstructural abnormalities have been shown to persist even in those with normalized LV size and EF.^9,10^ It is possible that improved phenotyping may help identify discriminating features of patients with recovered dilated cardiomyopathy (rDCM).

Diffusion tensor cardiovascular magnetic resonance (DT-CMR) is unique in offering in vivo dynamic assessment of the myocardial microstructure.^11^ It has provided insight into the wall thickening paradox whereby LV radial strain of around 40% exceeds what can be achieved by individual myocytes thickening of ≈8%. Reorientation of sheetlets (aggregated cardiomyocytes, which can be assessed using the DT-CMR parameter absolute angle of the second eigenvector of the diffusion tensor [E2A]) toward a more wall-perpendicular direction during systole facilitates wall thickening.^11^

The role of the microstructure in facilitating contractile reserve is unknown. In this study, we assess the microstructural response to low-dose dobutamine in healthy volunteers (HVols) and then compare the findings to patients with rDCM to examine for insights from novel diffusion biomarkers into the pathophysiology of remodeling.

## Methods

### Study Population

The data that support the findings of this study are available from the corresponding author on reasonable request. This study received approval from the National Research Ethics Committee (13/LO/1830), and all participants provided informed written consent. Patients with rDCM received their original diagnosis in accordance with diagnostic guidelines (World Health Organization) and required both LV end-diastolic volume normalization and a ≥10% increase in LV EF to ≥55%.^1^

### Image Acquisition

CMR was performed on a 3T scanner (Vida, Siemens, Erlangen, Germany). Data is presented from 3 stages: rest, 10 μg/kg per minute dobutamine, and at recovery (defined as blood pressure and heart rate [HR] within 25% of rest). Beta-blockers and caffeine were stopped for 48 hours before the scan to mitigate negative inotropy effects and thus their influence on DT-CMR parameters. Dobutamine was administered via a peripheral intravenous cannula with a long line extension to the infusion pump. HR and both systolic and diastolic blood pressures were measured at rest and every 2 minutes during dobutamine infusion. Dobutamine was infused for 3 minutes before image acquisition. Compressed sensing single breath-hold images were used to acquire the 3 LV long axes and a short-axis cine stack. Biphasic DT-CMR was performed as previously described.^11^ This protocol was used with the following modifications: 1 single mid-LV slice was imaged at a resolution of 3×3×10 mm with typically 4 averages at b=600 s/mm^2^ and, 1 reference average at b=150 s/mm^2^ and b=0 s/mm^2^. Sensitivity encoding (SENSE) parallel imaging was used with an acceleration factor of 2. Diastole is triggered as close to the R wave as possible, and systole is triggered to place the central k spaces lines at peak contraction. A beat-to-beat correction was applied to ensure that an HR corrected b value is used when calculating the diffusion tensor.

### Image Analysis

Volumes and EF were measured using CVI42 postprocessing software (Version 5.13.4, Circle Cardiovascular Imaging Inc, Calgary, Canada). Three-dimensional global radial, longitudinal, and circumferential strains were calculated using feature tracking.

Forthcoming references to E2A are intended to mean absolute values. For each patient, global E2A values were calculated as median E2A values over the LV myocardium in the slice imaged with DT-CMR at both diastole and systole. E2A mobility (reflecting sheetlet mobility) is the difference between global diastolic E2A and global systolic E2A. Biphasic mean E2A is the mean of the global diastolic and global systolic E2A values.

Mean diffusivity (MD) and fractional anisotropy were calculated as median values over the LV myocardium in the slice imaged with DT-CMR at both diastole and systole.

### Statistical Analysis

The Kolmogorov-Smirnov test and histograms were used to assess the normality of parameters and the evaluation of skewness. Baseline characteristics were compared with unpaired *t* tests, Mann-Whitney *U* test, or χ^2^ accordingly. Mixed effects models adjusted for age were used to estimate differences over time points and the interaction between time and group. All statistical tests were 2-tailed. Comparisons were made between groups, between rest and peak stress (10 μg/kg per minute dobutamine), and between rest and recovery. A level of significance was set at *P*<0.017 to take into account multiple comparisons as per the Bonferroni method. Data analysis was performed by Z.K. and medical statisticians using Prism version 9 and Stata (version 17.1, StataCorp, College Station, TX).

## Results

### Study Population

Twenty-two patients with rDCM and 35 HVols were recruited. Three HVols were excluded; 1 did not tolerate the scanner, and 2 had incidental findings. Two patients with rDCM were excluded due to relapse. Baseline characteristics are shown in Table 1. Patients with rDCM were older with a higher body mass index, had a lower mean LV EF 60±3% versus 67±6% (*P*<0.001), and lower absolute 3-dimensional global strain measures than HVols. There was no significant difference in the median BNP measurement between rDCM 29 ng/L (21–62) and HVol 26 ng/L (20–47); *P*=0.17.

**Table 1.**
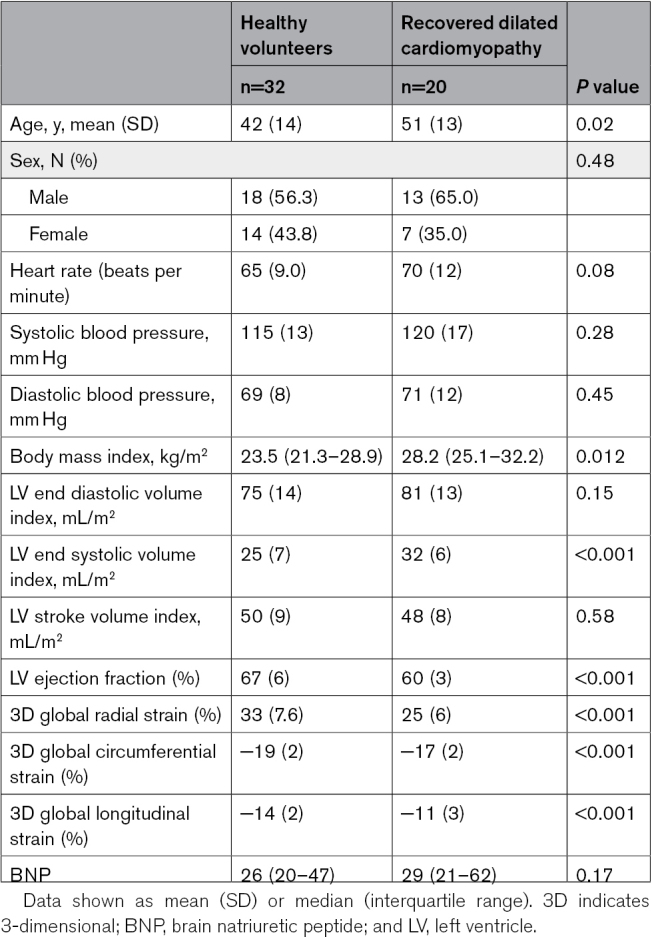
Demographics of Healthy Volunteers and Patients With Recovered Dilated Cardiomyopathy

The characteristics of the rDCM group are shown in Table 2. Median presenting LV EF was 22 (14%–30%) with 430 (368–640) days to recovery. All patients were taking at least 1 heart failure therapy and were classified as New York Heart Association I.

**Table 2.**
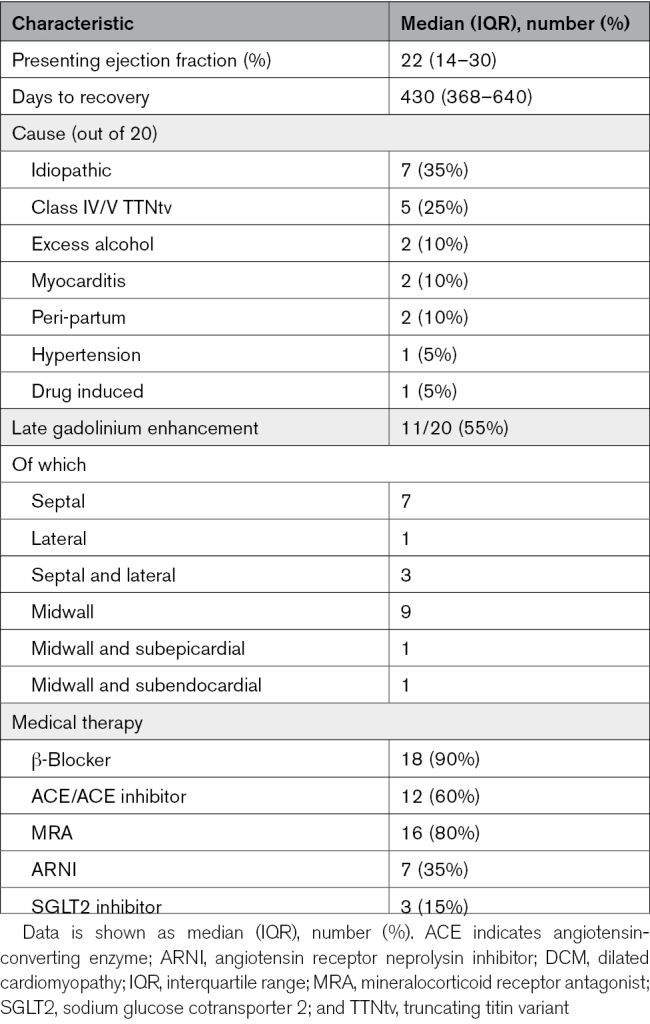
Characteristics of the Recovered DCM Group

### Dobutamine Stress Response in HVols

#### Hemodynamic and Strain Response

**Figure 1.**
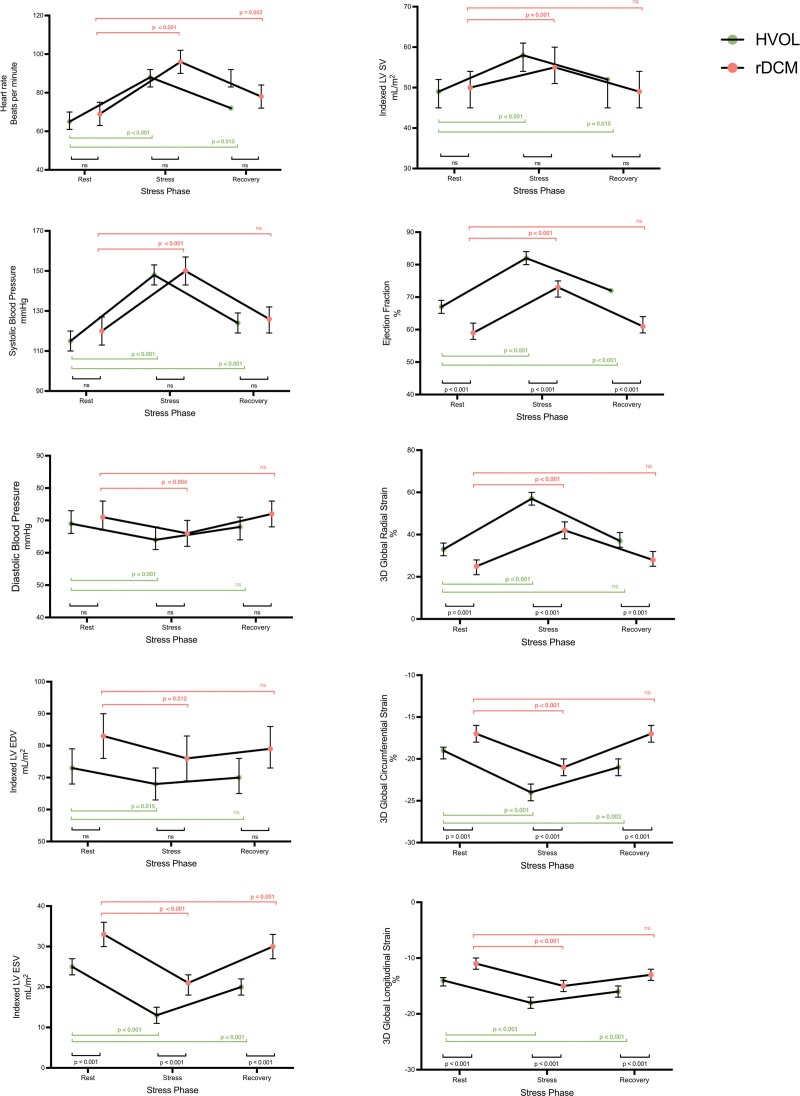
**Hemodynamic response to stress in healthy volunteers (HVols) and recovered dilated cardiomyopathy (rDCM).** Plots show estimated mean and 95% CIs at rest, peak stress, and recovery phases for both HVols (green) and patients with rDCM (red). Parameters at rest are compared with peak stress, and also with recovery as depicted by the horizontal bars with *P* values, shown in green for HVol and rDCM in red. Both HVol and rDCM increment heart rate, systolic blood pressure, indexed stroke volume (SV), ejection fraction, and strain parameters with stress. Bars and *P* values in black compare HVols with rDCM at rest, peak stress, and recovery. Ejection fraction and strain parameters are significantly reduced in rDCM compared with HVol. EDV indicates end-diastolic volume; ESV, end-systolic volume; and LV, left ventricle.

During stress, there was a significant increment in HR (65±9 bpm to 87±17 bpm; *P*<0.001), systolic blood pressure (115±13 to 148±16 mm Hg; *P*<0.001), indexed stroke volume (50±9 mL/m^2^ to 59±13 mL/m^2^; *P*<0.001) and LV EF (67±6% to 82±4%; *P*<0.001). Indexed LV end-diastolic volume reduced (75±14 mL/m^2^ to 72±17 mL/m^2^; *P*=0.015). The magnitude of global 3-dimensional radial, circumferential, and longitudinal strain also increased from rest to peak stress (all *P*<0.001). Changes in hemodynamic and strain parameters on stress and in recovery are shown in Figure 1; Table S1.

#### Microstructural Response in HVols

**Figure 2.**
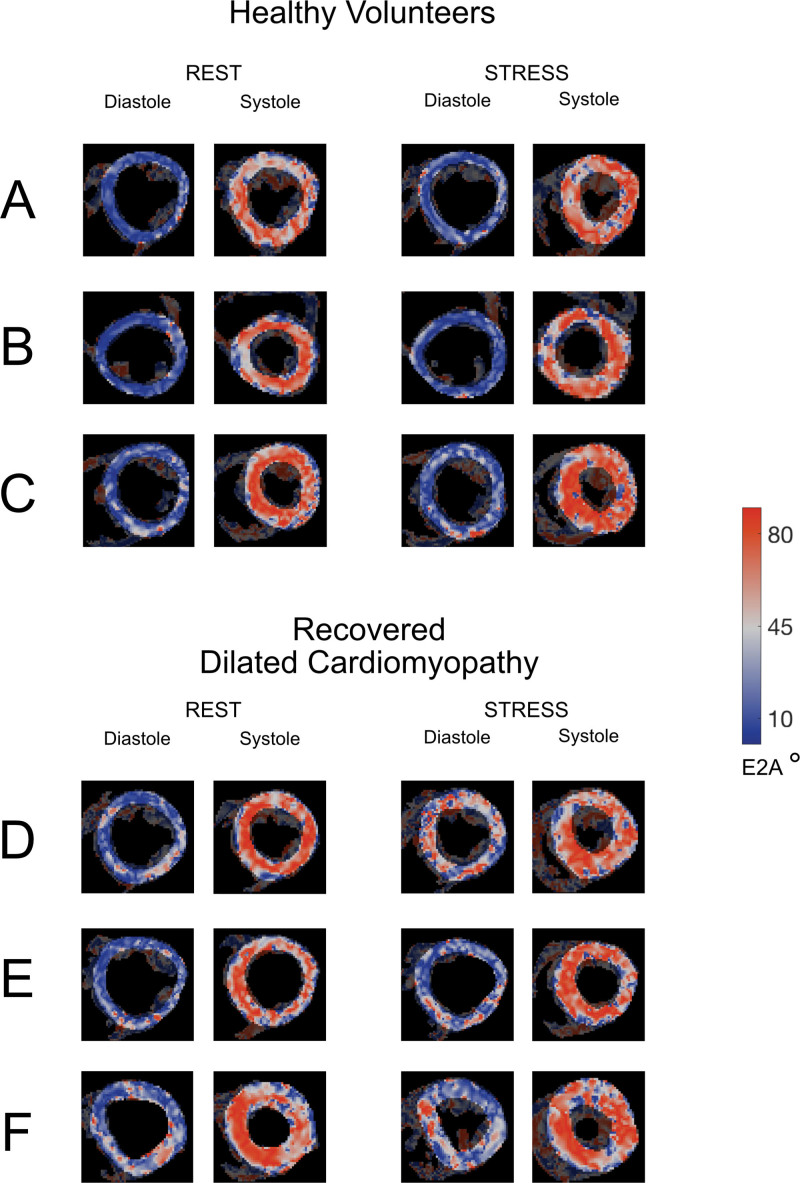
**Example diffusion tensor maps in healthy volunteers (HVols) and patients with recovered dilated cardiomyopathy (rDCM).** Example maps from 3 HVols (rows **A**, **B**, and **C**) and 3 patients with rDCM (rows **D**, **E**, and **F**) are shown. Maps show low E2A in blue (more wall-parallel aligned sheetlets) and high E2A in red (more wall-perpendicular aligned sheetlets). In health, at rest, sheetlets are predominantly wall-parallel in diastole (blue) and wall-perpendicular in systole (red). Under stress, both diastolic and systolic E2A increase, shown with more white and red areas in the diastolic maps and a greater preponderance of red in the systolic maps. In patients with rDCM at rest, while sheetlets are predominantly wall-parallel in diastole, the maps show areas of increased diastolic E2A in red. In systole, there is a widespread increase in E2A shown in red. Under stress, biphasic E2A is higher than at rest—the proportion of more radially oriented sheetlets in both diastole and systole increases, as shown by the greater amount of red in the maps.

**Figure 3.**
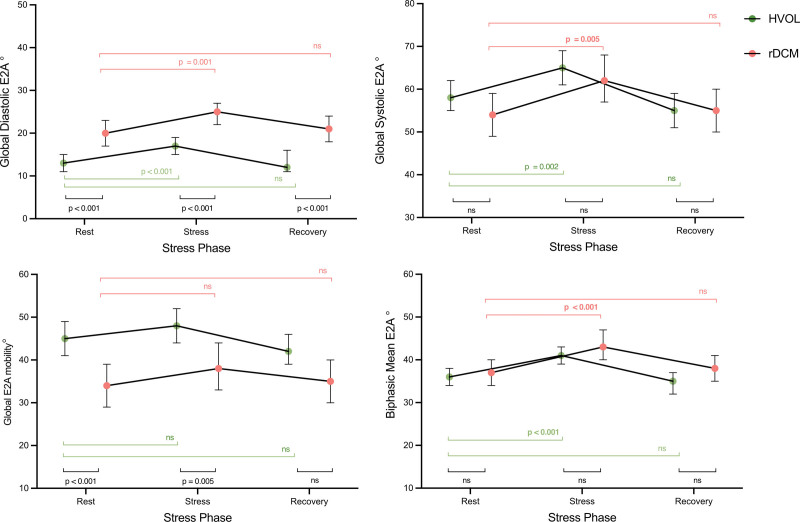
**Microstructural response to stress in healthy volunteers (HVols) and patients with recovered dilated cardiomyopathy (rDCM).** Plots show estimated mean and 95% CIs at rest, peak stress, and recovery phases for both HVols (green) and patients with rDCM (red). E2A parameters at rest are compared with peak stress, and also with recovery, as depicted by the horizontal bars with *P* values, shown in green for HVol and rDCM in red. All E2A parameters increase with stress, except E2A mobility. Bars and *P* values in black compare HVols with rDCM at rest, peak stress, and recovery. Diastolic E2A is higher in rDCM than HVol at all time points. E2A mobility is lower in rDCM at rest and also at stress.

**Figure 4.**
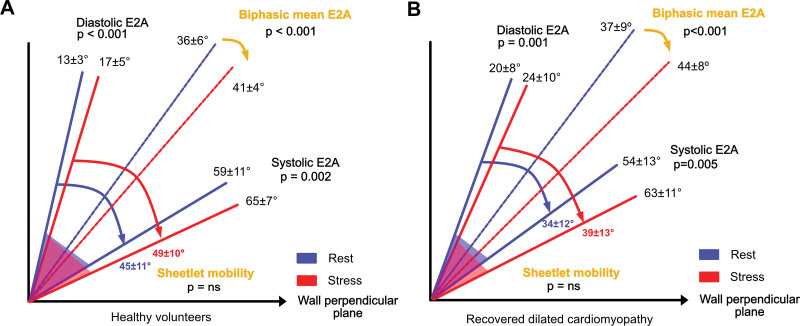
**Schematic of sheetlet dynamics during stress in healthy volunteers and recovered dilated cardiomyopathy (rDCM).** Sheetlets dynamics for the healthy volunteer group in **A** and for the rDCM group in **B**. Rest data are shown in blue and stress data in red. The rest plot shows that in diastole, sheetlets are primarily oriented within the wall-parallel plane (*y* axis) and move towards a more wall-perpendicular plane (*x* axis) in systole. At stress in red, the angle range over which sheetlets reorient (sheetlet mobility) remains similar in magnitude but has tilted further into the wall-perpendicular plane, as shown by increased biphasic mean E2A. This similar stress response is demonstrated in both health and rDCM.

Example diffusion tensor maps from an HVol are shown in Figure 2A through 2C, and E2A data for the HVol group are shown in Figure 3. Diastolic E2A at rest was 13±3°, rising to 17±5°at peak stress (*P*<0.001). In the recovery phase, diastolic E2A was 13±4°, similar to rest (*P*=0.92). Systolic E2A at rest was 59±11°, incrementing to 65±7° at peak stress (*P*=0.002). Systolic E2A in the recovery phase was similar to rest at 55±11° (*P*=0.085). E2A mobility was similar across phases: 45±11° at rest, 49±10° at peak stress, and 42±9° in recovery (*P*=0.19 and *P*=0.21, respectively). Biphasic mean E2A was 36±6° at rest, rising to 41±4° at stress, *P*<0.001. Recovery biphasic mean E2A was similar to rest at 35±6°, *P*=0.24. These changes are represented in schematic form in panel A of Figure 4 and within Table S2. MD and fractional anisotropy data are shown in Table S3 for reference. Biphasic MD increased during stress: diastolic MD from 1.19±0.07×10^−3^ mm^2^/s to 1.38±0.12×10^−3^ mm^2^/s; *P*<0.0001, and systolic MD from 1.05±0.08×10^−3^ mm^2^/s to 1.15±0.10×10^−3^ mm^2^/s; *P*<0.0001.

### Dobutamine Stress Response in Patients With rDCM

#### Hemodynamic and Strain Response

During stress, there was a significant increase in HR (70±12 bpm to 97±20 bpm; *P*<0.001), systolic blood pressure (120±17 mm Hg to 150±20 mm Hg; *P*<0.001), indexed stroke volume (48±18 mL/m^2^ to 53±14 mL/m^2^; *P*=0.001), and LVEF (60±3% to 73±7%; *P*<0.001). LV end-diastolic volume reduced at peak stress (81±13 mL/m^2^ to 73±17 mL/m^2^; *P*=0.012). The magnitude of global 3-dimensional radial, circumferential, and longitudinal strain also increased from rest to peak stress (all *P*<0.001). In the recovery phase, HR was still increased compared with rest. Changes in hemodynamic and strain parameters on stress and in recovery are shown in Figure 1; Table S2.

#### Microstructural Changes With Stress

Microstructural changes with stress are shown in example diffusion tensor maps in Figure 2 and E2A angle plots in Figure 3. In rDCM, diastolic E2A was 20±8° at rest, rising to 24±10° (*P*=0.001) at peak stress. In the recovery phase, diastolic E2A was 20±8°, similar to rest *P*=0.51. Systolic E2A at rest was 54±13°, rising to 63±11° at peak stress (*P*=0.005). In recovery, diastolic E2A was 55±13°, similar to rest (*P*=0.82). E2A mobility was 34±12° at rest, 39±13° at peak stress, and 35±13° in the recovery phase, but this phasic response was not significant (*P*=0.19 and *P*=0.74, respectively). Biphasic mean E2A was 37±9º at rest, rising to 44±8º at stress, *P*<0.001. Recovery biphasic mean E2A was similar to rest at 38±8º, *P*=0.45. Stress responses in rDCM are shown in schematic format in Figure 4B. MD and fractional anisotropy data are shown in Table S3 for reference. Biphasic MD increased during stress: diastolic MD from 1.25±0.09×10^−3^ mm^2^/s to 1.35±0.15×10^−3^ mm^2^/s; *P*<0.0001, and systolic MD from 1.13±0.09×10^−3^ mm^2^/s to 1.26±0.08×10^−3^ mm^2^/s; *P*<0.0001.

### Comparison of HVols With rDCM

#### Hemodynamic and Strain Response

At rest, peak stress, and recovery, HR, blood pressure, and indexed LV end-diastolic volume were similar between HVol and rDCM participants, as shown in Figure 1; Table S4. LVEF and all strain measures were significantly reduced in rDCM compared with HVols (all *P*<0.001).

#### Microstructural Differences Between Groups

Figure 3 shows the E2A differences between HVols and rDCM at both rest and stress. Diastolic E2A is significantly higher in the rDCM group: at rest, 20±8° versus 13±3°, *P*<0.001; peak stress, 24±10° versus 17±5°, *P*<0.001; and recovery, 20±8° versus 13±4°, *P*<0.001. Systolic E2A was similar in both cohorts at rest: rDCM 54±13° versus HVols 59±11° (*P*=0.19), peak stress 63±11° versus 65±7° (*P*=0.44), and recovery 55±11° versus 55±13° (*P*=0.99). E2A mobility was significantly lower in rDCM than the HVols at rest (34±12° versus 45±11°; *P*<0.001), at peak stress (39±13° versus 49±10°; *P*=0.005), but similar in recovery (35±13° versus 42±9°; *P*=0.026). Biphasic mean E2A was similar between groups at rest 37±9º versus 36±6° (*P*=0.56), at stress 44±8º versus 41±4° (*P*=0.28) and recovery 38±8º versus 35±6° (*P*=0.09).

**Figure 5.**
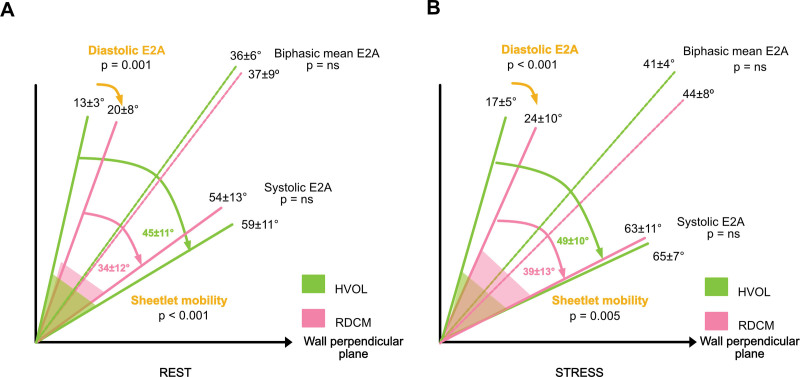
**Schematic diagram comparing sheetlet dynamics of healthy volunteers (HVols) and recovered dilated cardiomyopathy (rDCM).** Sheetlet dynamics are shown in green for HVols and red for patients with rDCM. The *x* axis represents the wall-perpendicular plane, and the *y* axis the wall-parallel plane. At rest in **A**, Patients with rDCM have greater diastolic E2A, and thus there is significantly reduced sheetlet mobility compared with HVols. The swivel segment (red triangle) is smaller and enclosed entirely by the green swivel segment of HVols. During stress in **B**, diastolic E2A remains increased compared with HVols. Sheetlet mobility does not increase to healthy levels and is significantly reduced.

When comparing rDCM versus the HVol cohort, there was no significant difference in the changes between rest and peak stress for diastolic E2A, systolic E2A, biphasic mean E2A, or E2A mobility. The differences in sheetlet dynamics between the 2 cohorts at rest and stress are shown schematically in Figure 5. The pink swivel segment of rDCM is completely enclosed with the green swivel segment of HVols at both rest and stress.

MD and fractional anisotropy data are shown in Table S5 for reference.

## Discussion

This is the first reported assessment of the dynamic microstructural response to stress in both HVols and patients with rDCM. Both cohorts demonstrated significant functional response to dobutamine stress with increased LV EF and strain. This data demonstrates that it is possible to acquire interpretable DT-CMR data that can show clinically meaningful differences between groups with just 4 breath-holds at a lower resolution, which in itself is an important result.

In health, dobutamine drives an increase in systolic E2A, so that sheetlets rotate further towards the wall-perpendicular plane on each contraction (Figure 4A). However, sheetlet mobility does not increase with stress, as might be expected. This may be because there is also an increase in diastolic E2A, which may reflect that the significant chronotropic response limits the time during which sheetlets can relax back to their resting orientation. Instead of increased sheetlet mobility, the range through which sheetlets cyclically reorient moves further towards the wall-perpendicular as shown by a higher biphasic mean E2A. This suggests that the contractility mediated by biphasic sheetlet reorientation has a limit, beyond which further reconfiguration into the wall perpendicular plane is required to access the cardiac reserve.

A similar pattern of fixed sheetlet mobility that moves further into the wall perpendicular plane is also seen in the rDCM response to stress (Figure 4B). On comparing rDCM response to HVols, it is clear that despite clinically significant reverse remodeling, there is persistence of microstructural abnormalities. In rDCM, resting diastolic E2A is increased compared with HVols, but systolic E2A is similar. This is interesting as it is the opposite of previously reported findings in patients with impaired DCM, where diastolic E2A is similar to HVols, but systolic E2A is markedly reduced.^11^ This may reflect an adaptive remodeling response, as there was no significant difference in resting HRs between the 2 groups to suggest impaired relaxation due to tachycardia. There was also a broad spread of diastolic E2A values in the rDCM cohort, which could reflect different remodeling responses; this could be important for understanding why different patients have different disease trajectories.

Although there are non-DT-CMR parameters that can discriminate HVols from patients with rDCM, such as reduced strain, DT-CMR additionally offers insight into the abnormalities of the microstructural apparatus. Patients with rDCM demonstrate reduced sheetlet mobility compared with HVols. This is in keeping with our previous work in which patients with rDCM with normal indexed LV end-diastolic volume and similar EF to HVols showed reduced sheetlet mobility.^10^ This reduction in resting sheetlet mobility is driven by the increased diastolic E2A.

During stress, rDCM microstructural impairment persisted, as sheetlet mobility did not increase to the level seen in HVols. While the biphasic mean E2A of rDCM at stress is higher than HVols, perhaps as a compensatory mechanism to account for the lower sheetlet mobility, this does not reach statistical significance, which may reflect our sample size. However, despite this, the study does identify that the resting DT-CMR is sufficiently sensitive to assess persistent microstructural abnormalities in the rDCM group. The increase in MD for both groups during stress likely reflects the sensitivity of DT-CMR in identifying the hyperemic response to dobutamine stress.

Sympathetic nervous system adaptation in heart failure by downregulation and desensitization of β-adrenergic receptors in patients with heart failure is well recognized.^12,13^ Studies have shown that while dobutamine promotes sarcomeric shortening, this can be variable, and that some dobutamine-treated cardiomyocytes can augment Ca2^+^ release without increasing contractility, and in some cells even decrease contractility.^14,15^ This may explain the broad variability in stress responses in our study. In addition, contractility is interlinked with preload and afterload, all of which have been altered with dobutamine stress, and this may also contribute to the spread of responses in our cohorts.

There may be several mechanisms through which the myocardium is able to increase contractility and promote recovery in DCM, bringing a range of therapeutic targets into view. A key focus of heart failure therapy has been catecholamine and neurohormonal pathways, but now interventions are exploring the structural mechanisms that underlie cardiac function.

In the forthcoming era of small molecule modulators of the contractile apparatus, which act through direct activation of the myosin molecules, cardiac reserve can be recruited through alternative pathways to calcium handling and cytosolic calcium accumulation. Cellular contraction studies have demonstrated wider variability in the degree of sarcomere shortening for dobutamine over omecamtiv mercarbil.^14^ Omecamtiv and danicamtiv can improve contractility, prolong left ventricular systolic ejection time, and increase calcium sensitivity, but this may come at the expense of diastolic function.^14,16–20^ With variable clinical responses to these small-molecule modulators, a targeted therapy approach to potential responders might be sought. DT-CMR has been histologically validated to show that changes in E2A correlate with changes in wall thickening.^11^ Therefore, this technique could be used to assess the effects of these myosin modulators and could yield interesting results to provide a potential way of dynamically tracking the structural response.

In patients with rDCM, there is a risk of relapse, which was demonstrated in the TRED-HF trial.^9,21,22^ Identifying those at risk of decompensation and further heart failure events is complex. Multi-scale deep phenotyping approaches to support risk stratification may be key. DT-CMR is the only tool available to phenotype the myocardium on this biological scale, capturing both the structure and dynamics on the microstructural scale. It is unclear why rDCM has significantly higher diastolic E2A than expected and whether the improved contractility of recovery comes at the expense of diastolic sheetlet function, which draws parallels with the myotrope studies.^14,16–20^ Furthermore, while not statistically significant, it is interesting that the biphasic mean E2A for rDCM under stress trends towards being higher than HVols, suggesting that an exaggerated wall perpendicular configuration of sheetlet mobility might be a compensatory measure for the resting lower sheetlet mobility. A prospective longitudinal approach in a larger cohort may help address these questions.

## Limitations

Study limitations include the size of the cohorts and their age difference, though the latter was adjusted for in comparisons. LVEF in the rDCM cohort is lower, although reflective of how recovery is often classified in real-life practice. Technical limitations of spatial and angular resolution mean that only the predominant sheetlet population and orientation are measured for each voxel. The relatively long diffusion time of stimulated echo acquisition mode (STEAM) sequences means that, while sensitivity does approach sheetlet scales (with water molecules diffusing ≈80 µm, spanning multiple cardiomyocyte cross sections), longer diffusion times may be optimal, and E2A effectively acts as an indirect microstructural measure of sheetlet orientation. Furthermore, there is HR variability (limited in part by the use of low-dose dobutamine), which can affect the b value, although prior work by our group has shown that these would be small.^23^

Although DT-CMR was limited to a single slice due to time limitations during dobutamine infusion, this approach has identified microstructural dysfunction in a number of pathologies.^11,24^

## Conclusions

This is the first report of the use of DT-CMR during dobutamine stress to assess the microstructural determinants of cardiac reserve in HVols and rDCM. This study shows that sheetlet mobility is reduced in patients with rDCM, which is driven by impaired diastolic sheetlet relaxation coupled with restored systolic sheetlet configuration, which is the opposite of the biphasic findings in acute DCM. Sheetlet mobility in both HVols and rDCM does not increase with stress, but accessing the cardiac reserve to increase contractility is facilitated by rotating the sheetlet swivel segment further into the radial plane. Furthermore, the reduced sheetlet mobility in patients with rDCM is unable to increase to levels seen in HVols.

Understanding the microstructural changes seen in health, the persistent abnormalities in rDCM at rest and stress may help identify those at risk of further relapse and guide our approach to prognostic therapies.

## Article Information

### Acknowledgments

The author would like to thank Rick Wage and Mr Simon Gover for their support with scan acquisition.

### Sources of Funding

This work was supported by a British Heart Foundation grant RG/19/1/34160/BHF.

### Disclosures

Dr Pennell is a stockholder and director of Cardiovascular Imaging Solutions and has received research support from Siemens. The other authors report no conflicts.

### Supplemental Material

Tables S1–S5

## Supplementary Material
