# Supplementary material for: Diffusion Tensor CMR Assessment of the Microstructural Response to Dobutamine Stress in Health and Comparison With Patients With Recovered Dilated Cardiomyopathy
Source: Circ Cardiovasc Imaging. 2025 Dec 12;19(2):e018226. doi: 10.1161/CIRCIMAGING.125.018226 (PMC12908641; doi:10.1161/CIRCIMAGING.125.018226)
Supplement: Supplementary file 1 [file hci-19-e018226-s001.pdf]

SUPPLEMENTAL MATERIAL

Table S1: Stress response of healthy volunteers

	Rest			Peak stress (10mg dobutamine)					Recovery				
	**N**	**Mean (SD)**	**Adjusted mean (95% CI)**	**N**	**Mean (SD)**	**Adjusted mean (95% CI)**	**Change from rest, Mean (95% CI)**	**Adj P**	**N**	**Mean (SD)**	**Adjusted mean (95% CI)**	**Change from rest, Mean (95% CI)**	**Adj P**
Heart rate (bpm)	32	65 (9)	65 (61,70)	32	87 (17)	88 (83,92)	22.5 (18.3,26.7)	<0.001	32	70 (11)	71 (83,92)	5.4 (1.2,9.7)	0.012
Systolic Blood Pressure (mmHg)	32	115 (13)	115 (110,120)	32	148 (16)	148 (143,153)	32.7 (28.7,36.7)	<0.001	32	124 (12)	124 (119,129)	8.9 (4.9,13.0)	<0.001
Diastolic Blood Pressure (mmHg)	32	69 (8)	69 (66,73)	32	64 (9)	64 (61,68)	-4.8 (-7.7, -1.8)	0.001	32	68 (8)	68 (64,71)	-1.5 (-4.3, 1.4)	0.32
LV End Diastolic Volume indexed (ml/m^2)	32	75 (14)	73 (68,79)	32	72 (17)	68 (63,73)	-5.3 (-9.6, -1.0)	0.015	30	74 (13)	70 (65,76)	-2.9 (-7.2, 1.5)	0.20
LV End Systolic Volume indexed (ml/m^2)	32	25 (7)	25 (23,27)	32	13 (5)	13 (11,15)	-11.9 (-13.6, -10.3)	<0.001	30	21 (7)	20 (18,22)	-4.5 (-6.1, -2.8)	<0.001
LV Stroke Volume indexed (ml/m^2)	32	50 (9)	49 (45,52)	32	59 (13)	58 (54,61)	9.0 (6.4, 11.6)	<0.001	30	53 (8)	52 (45,52)	3.3 (0.6, 6.0)	0.015
LV Ejection fraction (%)	32	67 (6)	67 (65,69)	32	82 (4)	82 (80,84)	15 (13, 17)	<0.001	30	72 (6)	72 (71,74)	5.8 (3.8, 7.7)	<0.001
3D GRS (%)	32	33 (8)	33 (30,36)	32	57 (13)	57 (54,60)	24 (20, 28)	<0.001	28	37 (7)	37 (34,41)	4 (-0.2, 8.0)	0.039
3D GCS (%)	32	-19 (2)	-19 (-20,-19)	32	-24 (2)	-24 (-25,-23)	-4.7 (-5.6, -3.8)	<0.001	28	-21 (2)	-21 (-22,-20)	-1.4 (-2.3, -0.5)	0.003
3D GLS (%)	32	-14 (2)	-14 (-15,-14)	32	-18 (3)	-18 (-19,-17)	-3.7 (-4.6, -2.8)	<0.001	28	-16 (2)	-16 (-17,-15)	-1.7 (-2.6, -0.8)	<0.001
Diastolic E2A (°)	32	13 (3)	13 (11,15)	31	17 (5)	17 (15,19)	3.7 (1.8,5.5)	<0.001	31	13 (4)	13 (11,16)	0.1 (-1.8,2.0)	0.92
Systolic E2A (°)	32	59 (11)	58 (55,62)	30	65 (7)	65 (61,69)	6.6 (2.4,10.7)	0.002	32	55 (11)	55 (51,59)	-3.6 (-7.6, 0.5)	0.085
E2A mobility (°)	32	45 (11)	45 (41,49)	29	49 (10)	48 (44,52)	3.0 (-1.5, 7.4)	0.19	31	42 (9)	42 (39,46)	-2.8 (-7.2, 1.6)	0.21
Biphasic mean E2A (°)	32	36 (6)	36 (34,38)	29	41 (4)	41 (39,43)	5.0 (2.9,7.2)	<0.001	31	35 (6)	35 (32,37)	-1.3 (-3.4, 0.9)	0.24

LV = left ventricle, GRS = global radial strain, GLS = global circumferential strain, GLS = global longitudinal strain
Adjusted mean and change from rest calculated using the linear mixed effect model, taking into account baseline age difference. P value given to 2sf

Table S2: Stress response of recovered dilated cardiomyopathy patients

	Rest			Peak stress (10mcg dobutamine)					Recovery				
	N	Mean (SD)	Adjusted mean (95% CI)	N	Mean (SD)	Adjusted mean (95% CI)	Change from rest, Mean (95% CI)	Adj P	N	Mean (SD)	Adjusted mean (95% CI)	Change from rest, Mean (95% CI)	Adj P
Heart rate (bpm)	20	70 (12)	69 (63,75)	20	97 (20)	96 (90,102)	27 (21.6, 32.4)	<0.001	20	79 (12)	78 (72,84)	8.6 (3.2,14.0)	0.002
Systolic Blood Pressure (mmHg)	20	120 (17)	120 (113,127)	20	150 (20)	150 (143,157)	30 (24.7,34.8)	<0.001	20	125 (17)	126 (119,132)	5.5 (0.37, 10.5)	0.036
Diastolic Blood Pressure (mmHg)	20	71 (12)	71 (67,76)	20	66 (11)	66 (62,70)	-5.5 (-9.2, -1.7)	0.004	20	72 (12)	72 (68,76)	0.7 (-3.1, 4.4)	0.73
LV End Diastolic Volume indexed (ml/m^2)	20	81 (13)	83 (76,90)	19	73 (17)	76 (69,83)	-7.0 (-12.5, -1.5)	0.012	20	77 (12)	79 (73,86)	-3.7 (-9.1, 1.7)	0.18
LV End Systolic Volume indexed (ml/m^2)	20	32 (6)	33 (30,36)	19	20 (7)	21 (18,23)	-12.6 (-15.0, -10.5)	<0.001	20	29 (5)	30 (27,33)	-3.4 (-5.5, -1.3)	0.001
LV Stroke Volume indexed (ml/m^2)	20	48 (8)	50 (45,54)	19	53 (14)	55 (51,60)	5.6 (2.2, 8.0)	0.001	20	48 (9)	49 (45,54)	-0.3 (-3.6, 3.0)	0.86
LV Ejection fraction (%)	20	60 (3)	59 (57,62)	19	73 (7)	73 (70,75)	13.2 (10.8, 15.6)	<0.001	20	62 (5)	61 (59,64)	2.2 (-0.1, 4.6)	0.063
3D GRS (%)	20	25 (6)	25 (21,28)	19	42 (8)	42 (38,46)	17 (12, 22)	<0.001	20	29 (7)	28 (25,32)	3.8 (0.9,8.5)	0.11
3D GCS (%)	20	-17 (2)	-17 (-18,-16)	19	-21 (3)	-21 (-22,-20)	-3.8 (-5.0, -2.7)	<0.001	20	-17 (2)	-17 (-18,-16)	0.07 (-1.1,1.2)	0.91
3D GLS (%)	20	-11 (3)	-11 (-12,-10)	19	20-15 (2)	-15 (-16,-14)	-3.7 (-4.8, -2.5)	<0.001	20	-13 (3)	-13 (-14,-12)	-1.3 (-2.4, -0.2)	0.026
Diastolic E2A (°)	20	20 (8)	20 (17,23)	15	24 (10)	25 (22,27)	4.4 (1.8,7.0)	0.001	17	20 (8)	21 (18,24)	0.8 (-1.6,3.3)	0.51
Systolic E2A (°)	20	54 (13)	54 (49,59)	15	63 (11)	62 (57,68)	8.1 (2.4,14.0)	0.005	18	55 (13)	55 (50,60)	0.6 (-4.7, 6.0)	0.82
E2A mobility (°)	20	34 (12)	34 (29,39)	14	39 (13)	38 (33,44)	4.2 (-2.0, 10.4)	0.19	17	35 (13)	35 (30,40)	0.98 (-4.8, 6.8)	0.74
Biphasic mean E2A (°)	20	37 (9)	37 (34,40)	14	44 (8)	43 (40,47)	6.2 (3.2,9.3)	<0.001	17	38 (8)	38 (35,41)	1.1 (-1.7,3.4)	0.45

LV = left ventricle, GRS = global radial strain, GLS = global circumferential strain, GLS = global longitudinal strain

Adjusted mean and change from rest calculated using the linear mixed effect model, taking into account multiple measurements and accounting for baseline age difference. P value given to 2sf

Table S3: Stress response of healthy volunteers and recovered dilated cardiomyopathy patients - mean diffusivity and fractional anisotropy

	Rest	Peak stress (10mcg dobutamine)			Recovery		
	**Adjusted mean (95% CI)**	**Adjusted mean (95% CI)**	**Change from rest, Mean (95% CI)**	**Adj P**	**Adjusted mean (95% CI)**	**Change from rest, Mean (95% CI)**	**Adj P**
**Healthy Volunteer**							
Diastolic FA	0.6 (0.59,0.61)	0.61 (0.60,0.62)	0.013 (0.0008, 0.025)	0.037	0.62 (0.60, 0.63)	0.016 (0.0037, 0.028)	0.011
Systolic FA	0.45 (0.43, 0.47)	0.47 (0.45, 0.49)	0.02 (0.0042, 0.037)	0.013	0.46 (0.44, 0.47)	0.007 (-0.0084, 0.023)	0.36
Diastolic MD (10^{-3} mm^2/s)	1.19 (1.16, 1.22)	1.38 (1.34, 1.41)	0.19 (0.147, 0.23)	<0.0001	1.21 (1.18, 1.25)	0.021 (-0.18, 0.06)	0.29
Systolic MD (10^{-3} mm^2/s)	1.05 (1.02, 1.09)	1.15 (1.11, 1.19)	0.99 (0.060, 0.14)	<0.0001	1.06 (1.02,1.20)	0.007 (-0.030, 0.44)	0.71
**Recovered DCM**							
Diastolic FA	0.59 (0.57, 0.60)	0.61 (0.60, 0.63)	0.027 (0.01, 0.044)	0.002	0.59 (0.58, 0.61)	0.0038 (-0.012, 0.020)	0.64
Systolic FA	0.47 (0.45, 0.49)	0.50 (0.48, 0.52)	0.033 (0.011, 0.054)	0.003	0.48 (0.46, 0.50)	0.012 (-0.0089, 0.034)	0.26
Diastolic MD (10^{-3} mm^2/s)	1.25 (1.21, 1.29)	1.35 (1.30, 1.40)	0.095 (0.041, 0.15)	<0.0001	1.25 (1.21, 1.30)	0.0016 (-0.059, 0.053)	0.95
Systolic MD (10^{-3} mm^2/s)	1.13 (1.09,1.18)	1.26 (1.21, 1.31)	0.13 (0.08, 0.18)	<0.0001	1.14 (1.09, 1.19)	0.0053 (-0.044, 0.05)	0.83

Data shown as mean (95% confidence intervals). MD = mean diffusivity, FA = fractional anisotropy. Adjusted mean and change from rest calculated using the linear mixed effect model, taking into account multiple measurements and accounting for baseline age difference. P value given to 2sf

Table S4: Comparison of stress responses in healthy volunteers and recovered dilated cardiomyopathy patients

	**Rest**				**Peak stress (10mg dobutamine)**				**Recovery**			
	**HVol**	**RDCM**			**HVol**	**RDCM**			**HVol**	**RDCM**		
	**Adj mean (95% CI)**	**Adj mean (95% CI)**	**Est mean difference (95% CI)**	**Adj p**	**Adj mean (95% CI)**	**Adj mean (95% CI)**	**Est mean difference (95% CI)**	**Adj p**	**Adj mean (95% CI)**	**Adj mean (95% CI)**	**Est mean difference (95% CI)**	**Adj p**
Heart rate (bpm)	65 (61,70)	69 (63,75)	4 (-3.6,11.8)	0.30	88 (83,92)	96 (90,102)	9 (0.9,0, 16.3)	0.03	71 (66,75)	78 (72,84)	7 (-0.4, 15.0)	0.064
Systolic Blood Pressure (mmHg)	115 (110,120)	120 (113,127)	5 (-3.7, 13.8)	0.26	148 (143,153)	150 (143,157)	2 (-6.7,10.8)	0.64	124 (119,129)	126 (119,132)	2 (-7.2,10.2)	0.73
Diastolic Blood Pressure (mmHg)	69 (66,73)	71 (67,76)	2 (-3.4,7.6)	0.46	64 (61,68)	66 (62,70)	1 (-4.1,7)	0.61	68 (64,71)	72 (68,76)	4 (-1.3,9.8)	0.13
LV End Diastolic Volume indexed (ml/m^2)	73 (68,79)	83 (76,90)	10 (1.2,18.3)	0.03	68 (63,73)	76 (69,83)	8 (-0.70,16.7)	0.07	70 (65,76)	79 (73,86)	9 (0.2,17.5)	0.045
LV End Systolic Volume indexed (ml/m^2)	25 (23,27)	33 (30,36)	8 (5.0,12.0)	<0.001	13 (11,15)	21 (18,23)	8 (4.3,11.4)	<0.001	20 (18,22)	30 (27,33)	10 (6.0,13.1)	<0.001
LV Stroke Volume indexed (ml/m^2)	49 (45,52)	50 (45,54)	1 (-4.5,6.5)	0.36	58 (54,61)	55 (51,60)	-2 (-8.0,3.1)	0.39	52 (45,52)	49 (45,54)	-3 (-8.1,2.9)	0.36
LV Ejection fraction (%)	67 (65,69)	59 (57,62)	-7 (-10.1, -4.3)	<0.001	82 (80,84)	73 (70,75)	-9 (-12.3,-6.4)	<0.001	72 (71,74)	61 (59,64)	-11 (-13.7,-7.8)	<0.001
3D global radial strain	33 (30,36)	25 (21,28)	-9 (-13.5,3.8)	0.001	57 (54,60)	42 (38,46)	-16 (-20.5,-10.6)	<0.001	37 (34,41)	28 (25,32)	-9 (-13.9, -3.97)	0.001
3D global circumferential strain	-19 (-20,-19)	-17 (-18,-16)	-2 (1.1,3.5)	<0.001	-24 (-25,-23)	-21 (-22,-20)	3 (1.9,4.4)	<0.001	-21 (-22,-20)	-17 (-18,-16)	4 (2.5,5.0)	<0.001
3D global Longitudinal strain	-14 (-15,-14)	-11 (-12,-14)	3 (1.5,4.4)	<0.001	-18 (-19,-17)	-15 (-16,-14)	3 (1.6, 4.5)	<0.001	-16 (-17,-15)	-13 (-14,-12)	3 (2.0,4.9)	<0.001
Diastolic E2A (°)	13 (11,15)	20 (17,23)	7 (3.3, 10)	<0.001	17 (15,19)	25 (22,27)	8 (3.9,11.2)	<0.001	13 (11,16)	21 (18,24)	8 (4.0,11)	<0.001
Systolic E2A (°)	58 (55,62)	54 (49,59)	-4 (-10,2.1)	0.19	65 (61,69)	62 (57,68)	-3 (-9.4,4.1)	0.44	55 (51,59)	55 (50,60)	0 (-6.4,6.5)	0.99
E2A mobility (°)	45 (41,49)	34 (29,39)	-11 (-17.3,-5.0)	<0.001	48 (44,52)	38 (33,44)	-10 (-16.8, -3.0)	0.005	42 (39,46)	35 (30,40)	-7 (-13.0,-0.9)	0.026
Biphasic mean E2A (°)	36 (34,38)	37 (34,40)	1 (-2.8,5.2)	0.56	41 (39,43)	43 (40,47)	2 (-1.9, 6.6)	0.28	35 (32,37)	38 (35,41)	4 (-0.5,7.7)	0.089

Data shown as mean (95% confidence intervals). LV = left ventricle. Adjusted mean and change from rest calculated using the linear mixed effect model, taking into account multiple measurements and accounting for baseline age difference. P value given to 2sf

Table S5: Comparison of stress responses in healthy volunteers and recovered dilated cardiomyopathy patients - mean diffusivity and fractional anisotropy

	**Rest**				**Peak stress (10mcg dobutamine)**				**Recovery**			
	**Healthy volunteer**	**rDCM**			**Healthy volunteer**	**rDCM**			**Healthy volunteer**		**rDCM**	
	**Adjusted mean (95% CI)**	**Adjusted mean (95% CI)**	**Estimated mean difference (95% CI)**	**Adj p**	**Adjusted mean (95% CI)**	**Adjusted mean (95% CI)**	**Estimated mean difference (95% CI)**	**Adj p**	**Adjusted mean (95% CI)**	**Adjusted mean (95% CI)**	**Estimated mean difference (95% CI)**	**Adj p**
Diastolic FA	0.6 (0.59,0.61)	0.59 (0.57, 0.60)	-0.013 (-0.032,0.0063)	0.19	0.61 (0.60,0.62)	0.61 (0.60, 0.63)	0.0016 (-0.019, 0.022)	0.88	0.62 (0.60, 0.63)	0.59 (0.58, 0.61)	-0.025 (-0.045, -0.0052)	0.013
Systolic FA	0.45 (0.43, 0.47)	0.47 (0.45, 0.49)	0.19 (-0.0094, 0.047)	0.19	0.47 (0.45, 0.49)	0.50 (0.48, 0.52)	0.031 (0.0011, 0.060)	0.042	0.46 (0.44, 0.47)	0.48 (0.46, 0.50)	0.024 (-0.0054, 0.052)	0.11
Diastolic MD (10^{-3} mm^2/s)	1.19 (1.16, 1.22)	1.25 (1.21, 1.29)	0.060 (-0.18, 0.12)	0.29	1.38 (1.34, 1.41)	1.35 (1.30, 1.40)	-0.030 (-0.092, 0.031)	0.33	1.21 (1.18, 1.25)	1.25 (1.21, 1.30)	0.041 (-0.018, 0.10)	0.17
Systolic MD (10^{-3} mm^2/s)	1.05 (1.02, 1.09)	1.12 (1.09,1.18)	0.08 (0.02,0.14)	<0.01	1.15 (1.11, 1.19)	1.26 (1.21, 1.31)	0.11 (0.04, 0.17)	0.001	1.06 (1.02,1.20)	1.14 (1.09, 1.19)	0.079 (0.02, 0.14)	0.014

Data shown as mean (95% confidence intervals). MD = mean diffusivity, FA = fractional anisotropy. Adjusted mean and change from rest calculated using the linear mixed effect model, taking into account multiple measurements and accounting for baseline age difference. P value given to 2sf
